# Discriminating Males and Unpredictable Females: Males Differentiate Self-Similar Facial Cues More than Females in the Judgment of Opposite-Sex Attractiveness

**DOI:** 10.1371/journal.pone.0090493

**Published:** 2014-03-03

**Authors:** Jin-Ying Zhuang, Sen Zhang, Jing Xu, Die Hu

**Affiliations:** Key Laboratory of Brain Functional Genomics (MOE & STCSM), School of Psychology and Cognitive Science, East China Normal University, Shanghai, China; East China Normal University, China

## Abstract

Attractiveness judgment in the context of mate preferences is thought to reflect an assessment of mate quality in relation to an absolute scale of genetic fitness and a relative scale of self-similarity. In this study, subjects judged the attractiveness and trustworthiness of faces in composite images that were manipulated to produce self-similar (self-resemblance) and dissimilar (other-resemblance) images. Males differentiated between self- and other-resemblance as well as among different degrees of self-resemblance in their attractiveness ratings; females did not. Specifically, in Experiment 1, using a morphing technique, we created previously unseen face images possessing different degrees (0%, 30%, 40%, or 50%) of incorporation of the subject's images (different degrees of self-resemblance) and found that males preferred images that were closer to average (0%) rather than more self-similar, whereas females showed no preference for any degree of self-similarity. In Experiment 2, we added a pro-social question about trustworthiness. We replicated the Experiment 1 attractiveness rating results and further found that males differentiated between self- and other-resemblance for the same degree of composites; women did not. Both males and females showed a similar preference for self-resemblances when judging trustworthiness. In conclusion, only males factored self-resemblance into their attractiveness ratings of opposite-sex individuals in a manner consistent with cues of reproductive fitness, although both sexes favored self-resemblance when judging trustworthiness.

## Introduction

Attractiveness judgment in the context of mate preferences describes the extent to which one individual is ‘attracted to’ or ‘drawn-in’ by another individual as a potential sexual partner. Attractiveness is fundamentally an index of mate quality, referring to an absolute scale of genetic fitness and a relative scale of self-similarity (shared genes) [1]. Physical symmetry, facial averageness, and sexual dimorphism are universal cues of attractiveness and the basic elements of the absolute scale. Symmetry signals good immuno-competence during development in spite of its inherent challenges[2], [3]. Facial averageness refers to the degree to which a given face resembles the majority of faces, the norm, within a given population. Evolutionary theories suggest that an average face is attractive because an alignment of features that is close to a population typicality is linked to genetic diversity [4], [5], which may result in less common proteins to which pathogens are poorly adapted. On the other hand, extreme (non-average) genotypes are more likely to be homozygous for deleterious alleles [5].

Features that highlight sexual dimorphism are related to hormone levels. In females, a more delicate bone structure, full lips, a small nose, and large eyes are rated as more feminine and attractive [6]; these features have been associated with higher levels of estrogen, and are thought to signal fecundity [7] and immuno-competence [8]. Conversely, characteristically male facial features, such as a square jaw, a heavier brow, and thinner lips are related to testosterone levels during development [9]. Testosterone is known to depress the immune system [10] and only those males with the best genes for immuno-competence should display these epigamic traits [11].

On the relative scale, similarity to the observer [12] generally enhances perceived attractiveness [13]–[16]. This phenomenon might be related to kin selection [17]. For example, in a study utilizing interactive financial investment games, DeBruine [18] found that people were more likely to trust those with whom they share a facial resemblance. Similarly, in a public good task, Krupp, DeBruine, and Barcay [19] found that people contributed more to the group when their group consisted of members whose faces resembled their own faces.

The coupling of physically similar individuals [20]–[25] is consistent with the notion of optimal out-breeding, wherein kin recognition may favor selection of phenotypically similar mates who were not raised in close proximity [26]–[28]. The purported benefit of optimal out-breeding is the maintenance of co-adapted genetic complexes [29] through the selection of a partner from the same population, who is thus likely to have appropriate adaptations for the local environment, or the enhancement of one's own genetic representation in future generations through the selection of a partner with some genetic matches [30]–[33].

Although both these absolute and relative scales may influence perceived attractiveness, researchers studying mating strategies agree that attractiveness ratings affect mate choice differently for males than for females. For example, even though sexually dimorphic characteristics signal absolute mate quality for both sexes, males' and females' responses to them are quite variable. Men reliably point to estrogen-linked feminine characteristics as attractive [6], whereas women's preference for highly masculinized male faces is inconsistent, with some studies demonstrating the trend clearly [34]–[36], and others not [9], [37], [38].

The relatively unstable preference by females for dimorphic features in males is usually interpreted as a reflection of how the female mating strategy differs from that of the male. Specifically, there are differences in reproductive constraints between the sexes [39], [40]. Male reproductive success skews toward competition for high fertile mates, whereas female reproductive success skews toward access to resources that affect fecundity, such as the quality of paternal care [41]–[44]. Proponents of trade-off theories of attractiveness judgments propose that the strength of women's attraction to men reflects not only the invariant facial characteristics, but also how women resolve cost-benefit trade-offs depending on their current mating strategy and social status [45]–[48]. These factors include the pursuance of short-term versus long-term relationships [49], women's judgments of their own attractiveness [50], [51], and women's own resources (e.g. income) and social statuses in the hierarchy [52].

Women's preferences for men with masculine facial characteristics appear to be influenced by several factors. A woman's preference for a masculine face is strongest when she nears ovulation [37], [53]–[55], suggesting an adaption to favor the genetic healthiness of her future offspring. Subsequently, as progesterone levels increase to prepare the body for potential pregnancy, the preference for a masculine face declines and women show enhancement of the self-similar preference and attraction to more androgynous male faces, perhaps reflecting increased preferences for caring, supportive, and trustworthy individuals.

Interestingly, the strength of women's attraction to masculine men also varies with regional differences in health- and violence-related factors [56]–[58]. Additionally, several neuroscientific studies have reported recently that women integrate information from social knowledge about a man into their judgment of his physical attractiveness. For example, Quist and colleagues [59] found that when pictured men were characterized as having a reputation for being faithful, masculine faces were judged to be significantly more attractive than androgynous male faces (produced through manipulation of the same source images); when the pictured men were characterized as having a reputation for being unfaithful, the masculinity-associated attractiveness edge eroded. A similar pattern of results was observed when unfaithful reputation condition was replaced with the social observation of the man having flirted with another woman while on a hypothetical date with the respondent [59].

Evidence suggests that the aforementioned sex differences in the judgment of attractiveness in relation to dimorphism in the context of mate choice—males' evaluation being consistent with fecundity cues, and females' judgment being unpredictable apparently related to attention to resources that affect fecundity—may extend to judgment of attractiveness in relation to cues of self-similarity. Kocsor, Rezneki, Juhász, and Bereczkei [60] found that while males preferred self-similar facial images (composite manipulated images) to dissimilar images matched for attractiveness, females did not show a significant preference.

**Figure 1:**
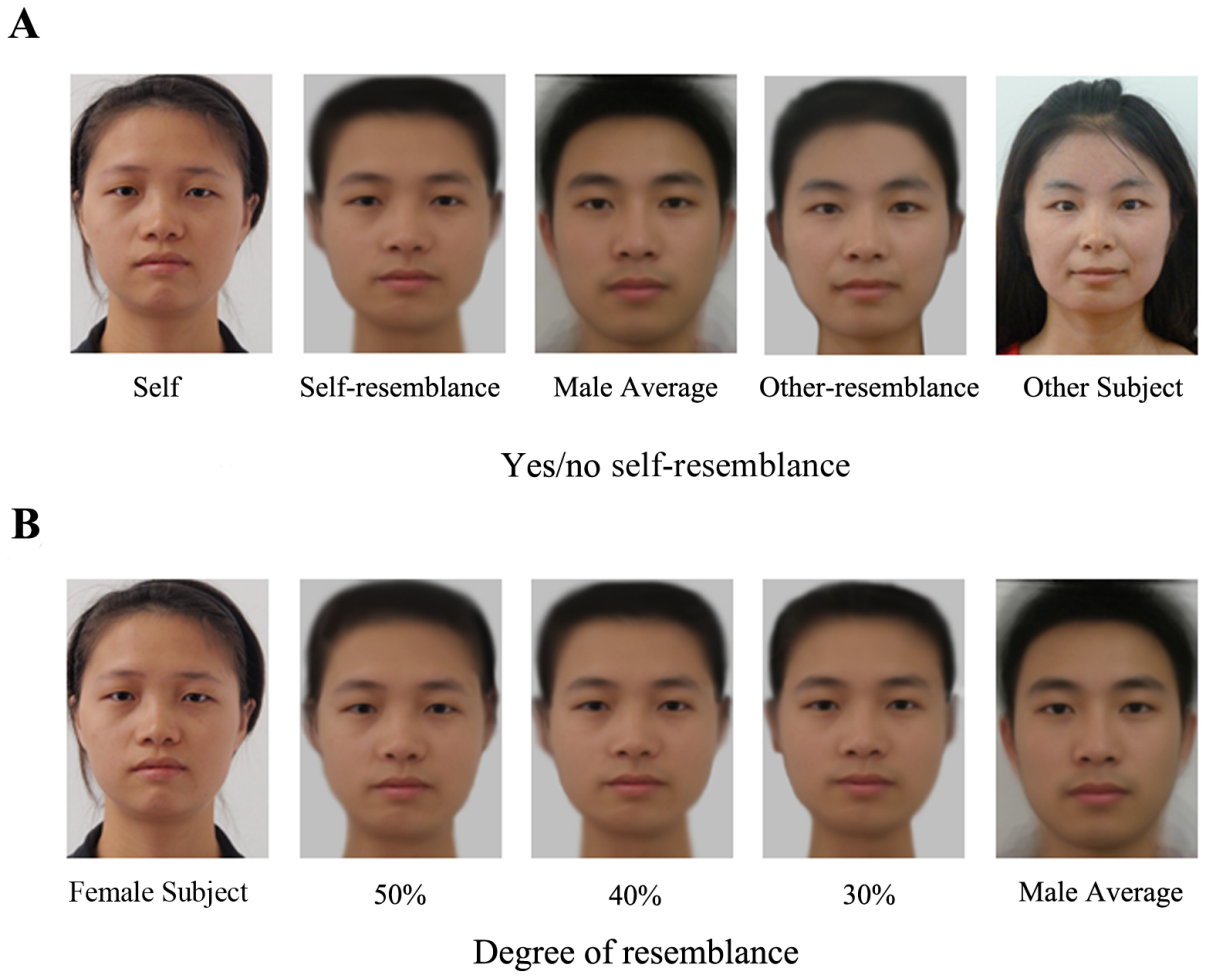
Production of face images. There were two kinds of computer graphic manipulations of face images: manipulation across different participants (A) and manipulation with the same participant (B). In the first instance (A), individual photographs of participants were morphed such that the self-similar image was a blend of a specified degree of that subject and the average composite (self-resemblance). One subject's self-resemblance was an other-subject blended image for another subjects. In the other instance (B), self-resemblance was varied to differing degrees to produce images with different degrees of self-similarity for each participant.

In this study, we investigated sex differences in the judgment of attractiveness of potential mates by manipulating facial cues of similarity. The images were manipulated to produce realistic composite images using a previously established technique [61], [62]. In the first condition, each individual participant's photograph was blended with an average composite image to a certain degree (e.g. 40% or 50%). This self-similar image (self-resemblance) to one subject was a blend dissimilar to the other subjects (other-participant-resemblance, other-resemblance for simplicity). We will call these kinds of image as the “yes/no self-resemblances” (Fig. 1A). The other one is manipulated with the same participants, that is, varying self-resemblances to a greater degree (e.g. 10%, 20%, 30%, 40%, etc.) to manifest the assessment of different distances of genetic relatedness with the same participant by showing the different degrees of similarity with that participant (see Fig. 1B). We will call it “degree of resemblances.” These graphic composites make it feasible to test the extent of subjects' discrimination of physical cues of similarity, and to compare male versus female self-similarity discrimination in mate selection.

## Experiment 1

In Experiment 1, we assessed males' and females' attractiveness ratings of opposite-sex faces with different degrees of self-similarity. We hypothesized that males would show greater discrimination for self-similarity than females.

### Materials and Methods

#### Participants

Fifty-four male (mean age  = 21.2 years, standard deviation [SD] = 1.13) and 54 female (mean age  = 20.9 years, SD = 1.45) undergraduate students from our university community participated in this study. Twenty-four participants of each sex were chosen randomly for making average composites and did not participate in the second phase of the experiment. The remaining 30 males and 30 females were divided into 10 same-sex groups of 6 subjects each. All participants within a group viewed the same set of composite photos. All participants were heterosexual and gave written informed consent. Subjects whose photographs have been published in Fig. 1 have given written informed consent, as outlined in the PLOS consent form, to publication of their photograph. The study protocol was approved by Ethics Committee of Shanghai Psychological Society.

#### Stimuli

More than two weeks before testing, a full-face color photograph of each participant was taken with a digital camera (Canon IXUS 700) under standardized diffuse lighting conditions against a constant background. The photographs were taken with a cover story that they would be used to develop a database of Chinese facial expressions to be used by several laboratories across China. They were asked to face the camera directly and to remove glasses (if worn), to pose with a neutral facial expression, and to pull hair away from their faces. The shape of each face in the photograph was delineated manually using 171 facial landmarks, and an average composite for each sex was created from 24 photographs each using established techniques [17], [61], [62] by combining the shape, color, and texture information from the individual images together. Next, with the same procedure, each participant's photograph and the average composite were delineated and a corresponding degree of shape difference (50%, 40%, 30%) between the participant and the same-sex average composite was applied to the opposite-sex average composite through a transformation technique. Thus, for each participant within a particular group, there were 3 opposite-sex self-resembling face composites (which we will refer to as self-resemblances for simplicity), 15 (5 other group members ×3 graded opposite-sex facial resemblances) opposite-sex other-resembling composites (called other-resemblances for simplicity), and 1 average opposite-sex composite (0% self-resemblance), totaling 19 composites per participant.

#### Procedure

More than two weeks after taking the photographs, participants were invited to return to the laboratory to finish the experiment. However, upon their return, they were directed to participate in an unrelated evaluation task. A short survey including queries about age, sex, sexual orientation, and relationship status was taken before testing. After the survey, all 19 composites were presented randomly one by one on a computer screen with the participant controlling the pace of presentation. After viewing each picture, the participant was asked, “How attractive do you think this person is?” Participants were asked to answer the question on a 1 (*not at all attractive*) to 7 (*very attractive*) Likert scale. The responses were analyzed with analyses of variance (ANOVAs), with a significance criterion of *p*<.05.

### Results and Discussion

**Figure 2:**
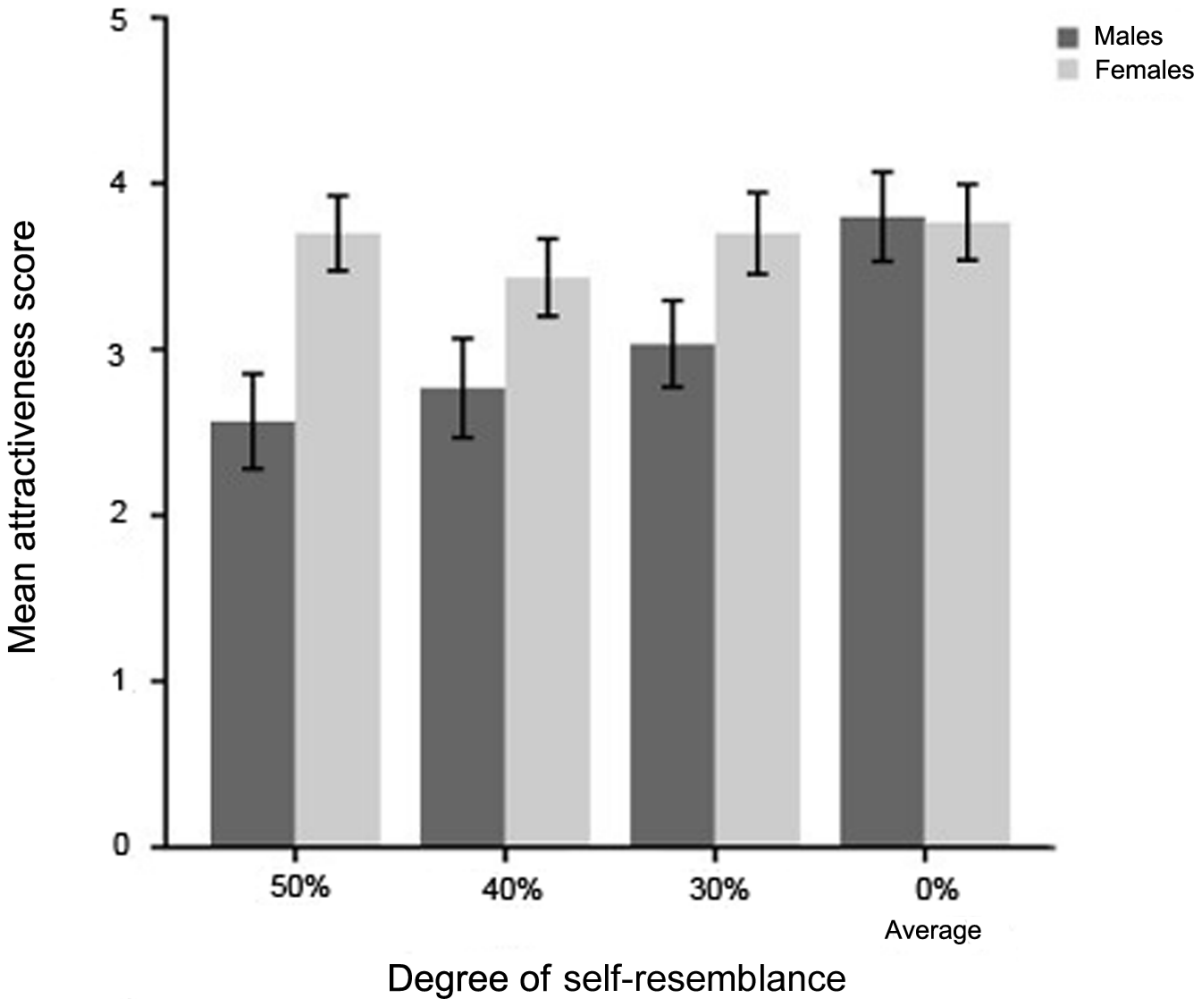
Mean attractiveness scores for different degrees of self-resemblance. Males found images with their own faces in them to be less attractive, with greater self-resemblance producing progressively lower attractiveness scores. Females rated attractiveness similarly across different degrees of self-similarity (error bars show 1 S.E.M.).

A mixed ANOVA of attractiveness scores with degree of self-resemblance (50%, 40%, 30%, 0%) as a repeated measures factor and sex of the participants (male, female) as the between-subjects factor showed significant main effects of the degree of self-resemblance [*F*(3, 174) = 4.92, *p* = .003, η*_p_^2^* = .078] and the participants' sex [*F*(1, 58) = 5.17, *p* = .027, η*_p_^2^* = .082]. There was a significant interaction between degree of self-resemblance and participants' sex, *F*(3, 174) = 2.87, *p* = .038, η*_p_^2^* = .047. As shown in Figure 2, simple effects analysis yielded a significant effect of degree of self-resemblance for males, *F*(3, 174) = 7.24, *p*<.001, η*_p_^2^* = .11, but not for females, *F*(3,174) = 0.54, *p* = .65, η*_p_^2^* = .009. Furthermore, *post hoc* tests (Bonferroni) revealed significant differences between many resemblance-degrees and the average composite for male participants (0% vs. 30%, *p* = .039; 0% vs. 40%, *p* = .015; 0% vs. 50%, *p* = .012).

These results confirmed our hypothesis that males differentiate degree of self-similarity more than females. Specifically, males not only found self-resemblances to be less attractive than an average composite, but the more of themselves that was in the composites, the less attractive male participants judged them to be. Meanwhile, women preferred images that contain different percentages of their own face roughly ‘equally’ (see Fig. 2). These findings suggest that in the process of mate choice, men may avoid inbreeding actively. Our finding that males rated the average composite as the most attractive face is a replication of the ‘average effect’ and provides evidence that males may be acting on an ability to select for desirable genetics [4], [5] when choosing a mate. There was no evidence of this behavior in women.

## Experiment 2

If the gender-specific self-resemblance discriminating effect found in Experiment 1 reflects the adaptive results of different mating strategies of males and females, the preferences of women and men should concur in other social contexts, such as in the context of cooperation. Therefore, in Experiment 2, we replicated the method of Experiment 1, but extended our test to the context of cooperation. We predicted that, in the context of mate choice, the attractiveness judgments of males and females would differ significantly from each other as they did in Experiment 1, and further hypothesized that we would not see this effect of sex of the observer on evaluations of trustworthiness, an index of pro-social behavior [14], [18], [19].

### Materials and Methods

#### Participants

A total of 76 (38 males and 38 females) undergraduate and graduate students from the university community (mean age  = 21.4 years, range, 19–27 years) participated in this study. We randomly chose 16 of these participants (8 males, 8 females) for making average composites, and they did not take part in the second phase of the experiment. The remaining 60 participants (30 males, 30 females) were divided into 12 same-sex groups of 5 students each. All participants in a group viewed the same set of composites. All participants were heterosexual and gave written informed consent.

#### Stimuli

More than two weeks before testing, a full-face color photograph of each participant was taken with a digital camera (Canon IXUS 700) under standardized diffuse lighting conditions against a constant background. The cover story and the requirement for participants to be photographed was the same as in Experiment 1. Next, male and female facial average composites were respectively created by morphing 8 same-sex individual photographs together using the same methods as in Experiment 1. Each participant's photograph was morphed into six different degrees of self-resemblance (60%, 50%, 40%, 30%, 20%, 10%) with the average opposite-sex composite. Thus for each participant within a particular group, there were 6 opposite-sex self-resemblances, 24 (4 other group members ×6 graded opposite-sex facial resemblances) other-resemblances, and one opposite-sex average composite (0% self-resemblance), totaling 31 composites per participant.

#### Procedure

As in Experiment 1, more than two weeks after taking the photographs, participants were invited to return to the laboratory to finish the experiment, but were directed to participate in an unrelated evaluation task. After completion of an informational survey (same as in Experiment 1), all 31 composites were presented randomly one by one on a computer screen with the participant controlling the pace of presentation. After viewing each picture, participants were asked to answer two questions: “How attractive do you think this person is?” and “Is this person trustworthy?” They were directed to answer the questions on a 1 (*not at all attractive/trustworthy*) to 7 (*very attractive/trustworthy*) Likert scale. The order of the presentation of these questions was counterbalanced within each participant. At the completion of the experiment, participants were encouraged to comment on any aspect of the study. None of the participants reported correctly that the images they viewed contained information from their own faces. The statistical methods used in Experiment 1 were also used in Experiment 2.

### Results and Discussion

**Figure 3:**
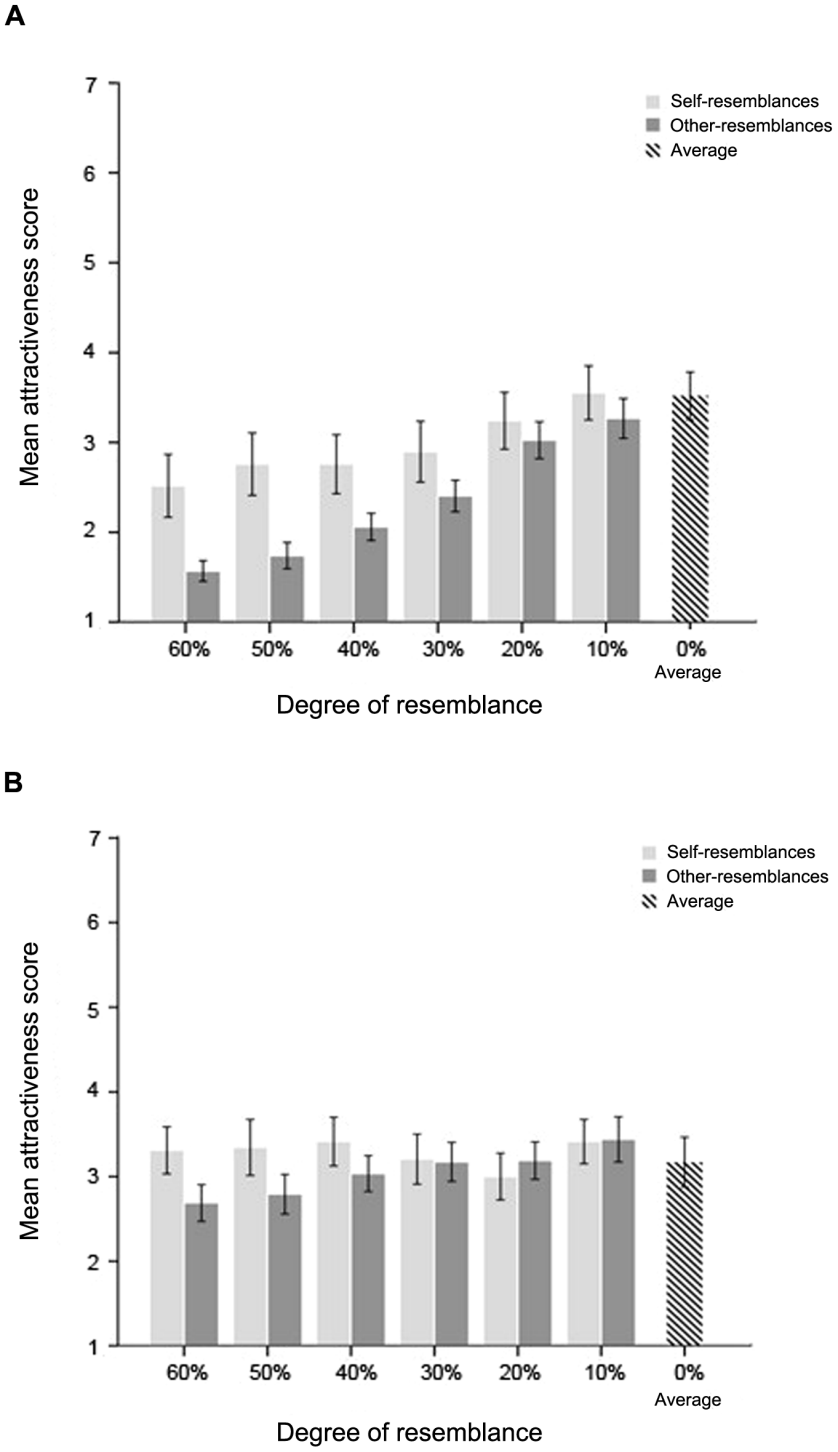
Mean attractiveness scores for self-resemblance, other-resemblance, and average composite, graphed in relation to degree of resemblance. (A) Males showed a clear preference for proximity to the average composite, but preferred self-resemblances to other-resemblances for the same degree of resemblance. (B) Females did not show preferences in relation to degree of self-resemblance or self-resemblance versus other-resemblance of the same degree (error bars show 1 S.E.M.).

**Figure 4:**
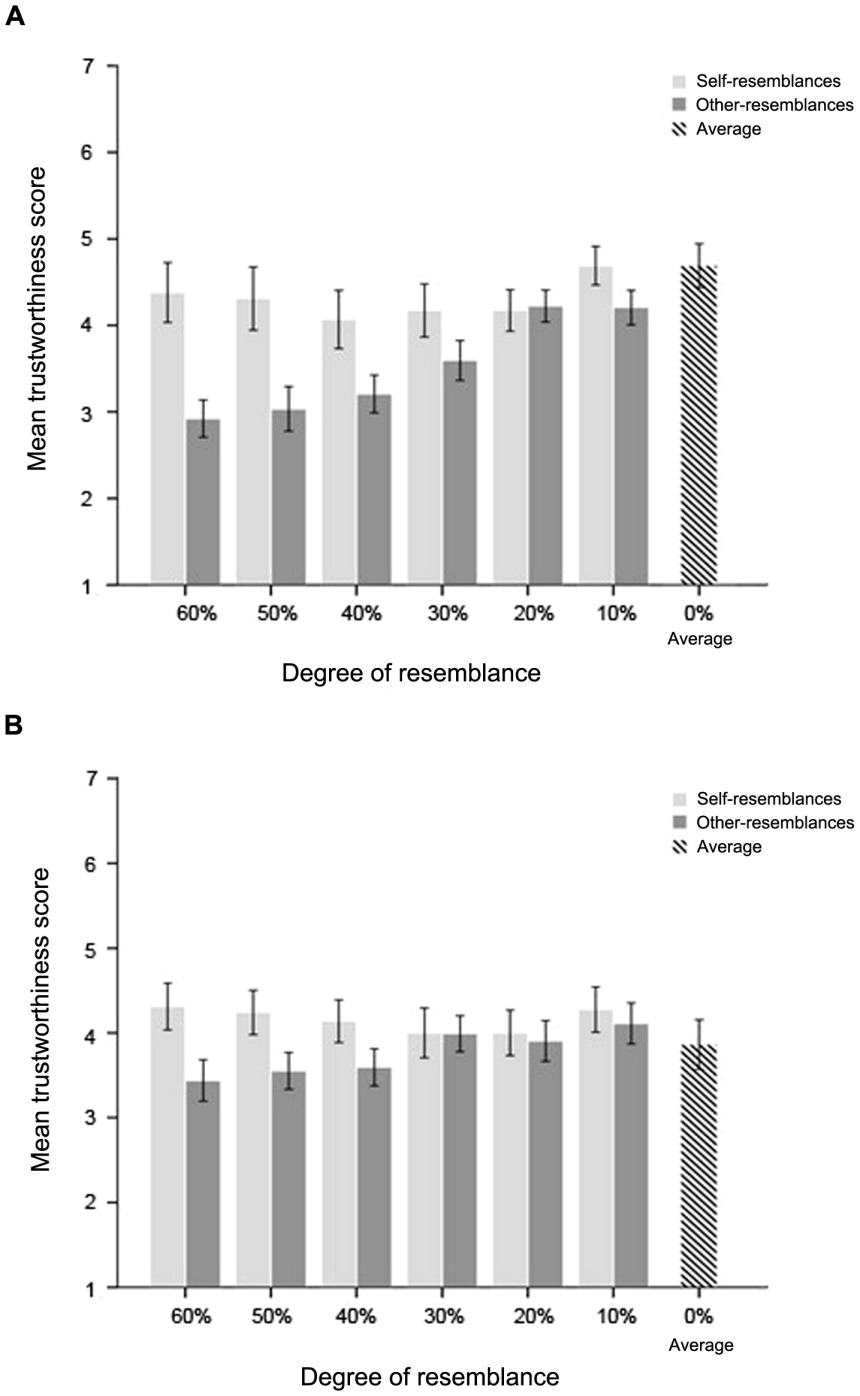
Mean trustworthiness scores for self-resemblance, other-resemblance, and average composite, graphed in relation to degree of resemblance. In the context of kinship cooperation, both males (A) and females (B) trusted the different degrees of self-resemblances roughly equally, and both groups distrusted other-resemblances more, the closer to the individualized pole they were (error bars show 1 S.E.M.).

Data from one male and one female participant were excluded due to missing data points. The mean scores of both ratings for each degree of self-resemblances and other-resemblances and the opposite-sex average composite by the same participants were calculated and graphed relative to resemblance degree (Figs. 3 and 4).

#### Attractiveness

A mixed ANOVA of attractiveness scores with degree of resemblance (60%, 50%, 40%, 30%, 20%, 10%) and yes/no self-resemblances (self-resemblance, other-resemblance) as repeated measures factors and the sex of participants (male, female) as the between-subjects factor showed significant main effects of degree of resemblance [*F*(5, 280) = 13.82, *p*<.001, η*_p_^2^* = .20] and yes/no self-resemblance [*F*(1, 56) = 13.73, *p*<.001, η*_p_^2^* = .20]. Participants rated images that were close to average (10% degree of resemblance) as the most attractive (*M* = 3.42) and preferred the self-resemblances (*M* = 3.12) to other-resemblances (*M* = 2.70). The main effect of participants' sex was close to significant, *F* (1, 56) = 3.29, *p* = .075, η*_p_^2^* = .055. The interactions between degree of resemblance and sex of participants [*F*(5, 280) = 6.17, *p*<.001, η*_p_^2^* = .10] and between degree of resemblance and yes/no self-resemblance [*F*(5, 280) = 5.14, *p*<.001, η*_p_^2^* = 0.08] were also significant. Simple effects analysis showed a significant effect of degree of resemblance for male participants, *F*(5, 280) = 18.49, *p*<.001, η*_p_^2^* = .25, but not for female participants, *F*(5, 280) = 1.51, *p* = .19, η*_p_^2^* = .026. Our analysis of the interaction between degree of resemblance and yes/no self-resemblance indicated that self-resemblances (60%, 50%, and 40%) were rated consistently as more attractive than non-resemblances (Fig. 3).

A subsequent mixed ANOVA of attractiveness score of the three most distinctive image types (60% self-resemblance, 60% other-resemblance, and the average composite) with the sex of participants and resemblance type as independent factors showed a significant main effect of image type, *F*(2, 112) = 21.56, *p*<.001, η*_p_^2^* = .28. The main effect of participants' sex was close to significant, *F*(1, 56) = 2.99, *p* = .09, η*_p_^2^* = .051. There was a significant image type × participant sex interaction, *F*(2, 112) = 8.40, *p*<.001, η*_p_^2^* = .13. An analysis within each sex indicated that males differentiated according to both degree of resemblance and yes/no self-resemblance, with the average composite getting the highest attractiveness score (*M* = 3.52), the 60% other-resemblance getting the lowest score (*M* = .57), and the 60% self-resemblance getting an intermediate score (*M* = 2.52). Females, in contrast, did not differentiate significantly between these three composites.

#### Trustworthiness

A mixed ANOVA of trustworthiness ratings with degree of resemblance and yes/no self-resemblance as repeated measures factors and sex of participants as the between-subjects factor showed significant main effects of degree of resemblance [*F*(5, 280) = 7.66, *p*<.001, η*_p_^2^* = .12] and yes/no self-resemblance [*F*(1, 56) = 30.65, *p*<.001, η*_p_^2^* = .35)]. There was not a significant main effect of the subjects' sex, *F*(1, 56) = 0.029, p = .87, η*_p_^2^* = .001. There was a significant interaction between degree of resemblance and yes/no self-resemblance, *F*(5, 280) = 8.47, *p*<.001, η*_p_^2^* = .13. A follow-up simple effects analysis revealed significant differences among other-resemblances by degree, *F*(5, 280) = 26.68, *p*<.001, η*_p_^2^* = .32, but not among self-resemblances by degree, *F*(5, 280) = 1.49, *p* = .195, η*_p_^2^* = .026, indicating that participants trusted the different degrees of self-resemblance roughly equally, but distrusted other-resemblances more, the more individualized the images were (Fig. 4).

The results of Experiment 2 replicated the results of Experiment 1 in that males exhibited greater differentiation than females in acting on facial cues of different degrees of self-similarity. In addition, males preferred self-resemblances to other-resemblances across several degrees of individualized images. Although there was a trend for women to prefer self-resemblances to other-resemblances, the effect was not significant. If yes/no self-resemblance cue discrimination indicates a favoring of optimal out-breeding, our results point to an important role of males in the process of optimal out-breeding. In the evaluation of trustworthiness, the responses of men and women were similar, with both showing equal preference for any image containing their own faces, regardless of degree. Hence, judgments related to cooperative interactions were treated distinctly from those related to mate choice interactions.

## Discussion

In the judgment of attractiveness of opposite sex faces with reference to the relative scale (similarity), this study replicated the sex effect found previously in an evaluation context with reference to an absolute scale. That is, males' evaluations of the attractiveness of potential mates was consistent with physical cues of reproductive fitness, in terms of expression of an average phenotype for one's population and lacking strong self-similarity, whereas females' evaluations were not. Specifically, for facial cues with different degrees of self-resemblance (representing varying genetic distance from the observer), men rated the facial composite with least degree of self-similarity as the most attractive and rated the most individualized composite with the greatest degree of self-similarity (60%) as the least attractive, with a linear gradation between these extremes. On the other hand, women's ratings appeared to be independent of degree of self-resemblance. Men showed active avoidance of inbreeding in mate choice, but no evidence of such avoidance was apparent for women.

Male subjects preferred self-resemblance generally to other-resemblance for the same degree of composite (e.g. 60%, 50%, 40%), replicating the ‘self-preference effect’ [13]–[16] and providing support for the theory of optimal out-breeding. Women did not show this self-resemblance preference.

Our finding of males rating the average facial composite as the most attractive, and the most individualized composites as the least attractive, with a linear individualization-related trend between these poles, is a replication of previous work by Langlois and Roggman [63] who showed that an averaged face was more attractive than individualized original faces. If averageness is linked to genetic diversity [4], [5], as has been supposed, then males in our study showed adeptness in detecting facial cues related to desirable genes.

In summary, in the context of mate choice, males' judgment of attractiveness of potential mates with the relative scale is consistent with physical cues of reproductive fitness by showing a trend of inbreeding avoidance and optimal out-breeding, as well as a sensitivity to cues of good genes, but women did not show these behaviors. Other animals have also been reported to show this kind of sex difference in mate choice. For example, despite a low mating investment and a male-biased operational sex ratio, male German cockroaches (*Blattella germanica*) were observed to court non-sibling females preferentially [64]; females, in turn, mated with the most vigorously courting males [65]. Males of two sympatric species of live-bearing fish, *Gambusia affinis* and *Gambusia geiseri*, contributed to sexual isolation by mating preferentially with conspecific females, while females did not show a preference for conspecific males over heterospecific males [66].

Studies of assays of sperm number and viability demonstrated that males in several species adjusted their investment in ejaculates in relation to their mating status and/or the reproductive fitness of individual females (reviewed in [67]; see also [68]–[71]). *Cordylochernes scorpioides* males exhibited a remarkable ability to distinguish between virgin, once-mated, and multiply-mated females using olfactory cues deposited on the female by other males during mating, and allocated their sperm accordingly, with virgin females receiving nearly three times the sperm as females exposed to three previous males, presumably in alignment with the risk of sperm competition [72]. Likewise, male fiddler crab (*Uca mjoebergi*) spend more time courting and direct more waves at larger females, an apparent adaptation to the fact that, in this species, female body size correlates positively with fecundity [73]. However, although there is prior evidence supporting this claim from other animals, we must be very careful in making any direct behavioral comparisons across highly divergent species. More research is needed to probe sexual differences in discriminating behaviors related to reproductive fitness in humans and to investigate the mechanisms underlying the differences.

The results of this study seem a bit counter-intuitive since females in most species have been long considered the “choosy” party [40], [74], [75]. The lack of discrimination in cues of self-similarity by females might reflect the involvement of other factors in women's preference for mates. For example, women's preference for self-resembling faces has been shown to be influenced by their menstrual cycles [15], with greater preference for self-resemblances during the luteal phase than during the fertile phase, perhaps due to the motivation to seek support from kin when pregnancy is possible. The lack of a control for women's menstrual cycles, as well as other factors, such as the short versus long-term mating strategy, individual life history, etc., may have contributed to the non-significant results for women in this study.

However, studies have indicated that males' preference for self-resemblances may also be context dependent. For example, under stressful conditions, males shifted their preferences from self-similar to dissimilar mates [76]. In addition, both males and females downgraded self-resembling mates with a short-term strategy relative to a long-term strategy [77]. Growing evidence from other animals suggest that male choosiness is more common than expected [78]–[81]. Hence, future research should investigate factors that affect male and female mate preferences differentially to explore gender-specific roles in the context of mate choice.

This study had another noteworthy limitation. That is, the sexually dimorphic characteristics of the composites could be a confounding factor. However, the different degrees of other-resemblance added in Experiment 2 could act as a control factor to exclude the influence of the sexual dimorphism, as long as we were concerned only with sex differences in the judgment of attractiveness of same-degree-of-resemblance between self-resemblances and other-resemblances. Second, sexual dimorphism increased linearly from the most individualized images to the average composites, and self-resemblance degree was a covariate with facial attractiveness and thus could be a baseline for inferences about the discriminating behaviors of males and females.

In conclusion, this study was the first to test the hypothesis that human males incorporate cues of facial similarity related to reproductive fitness into their attractiveness judgments more than females. Although both males' and females' mate preferences may be context dependent, under natural (non-manipulated) conditions, this study replicated the sex differences found in previous researches of sexual dimorphism. That is, males' attraction to potential mates was consistent with their being responsive to established cues of reproductive fitness, whereas, females' preferences were unpredictable.
